# Spatial Demonstratives and Perspective Taking in English and Japanese

**DOI:** 10.1111/cogs.70183

**Published:** 2026-03-23

**Authors:** Harmen B. Gudde, Jacqueline Collier, Kenny R. Coventry

**Affiliations:** ^1^ Helmholtz Institute Department of Experimental Psychology, Utrecht University; ^2^ School of Psychology University of East Anglia; ^3^ Hanse‐Wissenschaftskolleg/Hanse‐Institute for Advanced Study Delmenhorst

**Keywords:** Spatial demonstratives, Perspective taking, Cross‐linguistic variation, Spatial cognition

## Abstract

There is much debate regarding the extent to which languages express the same spatial parameters or whether spatial communication is essentially diverse. In this paper, we explore “perspective taking” in spatial demonstrative systems as a means of exploring between and within language variation. We test the effects of egocentric distance and addressee position on demonstrative production in speakers of two languages with two purportedly different demonstrative systems: English and Japanese. We find that speakers of both languages show perspective taking in their demonstrative use, with an overall increase in perspective taking in both languages when there was greater interaction between participants during the experimental task. We propose a framework unifying different theoretical accounts of demonstrative systems in which speakers of both languages choose a spatial reference frame prior to selecting from the available demonstrative terms in their language. Such an approach accounts for diversity while maintaining the same underlying processes between languages.

## 1 The expression of space across languages

Spatial language occupies a key place in debates regarding the relationship between language and mind. On the one hand, it has been argued that languages are structured similarly vis‐à‐vis spatial distinctions reflecting the shared perceptual apparatus and communicative needs of people across cultures (Clark, 1983). On the other hand, it has been argued that there is cross‐linguistic variation in spatial communication systems with languages carving up space in fundamentally different ways (Majid, Bowerman, Kita, Haun, & Levinson, 2004). Perhaps the best‐known example of such variation is focused on spatial reference frames across languages. While Indo‐European languages (e.g., English) tend to describe space most from the point of view of the speaker (the egocentric perspective: “*The cup is to the right of the teapot*”—on the right side from the speaker's perspective), languages such as Tzeltal generally prioritize allocentric, geocentric, or absolute relations over egocentric space (e.g., “*The cup is downhill/south of the teapot*”). Here, we shed light on this debate, focusing on (arguably) the most fundamental class of spatial terms across languages (see Diessel & Coventry, 2020 for discussion), spatial demonstratives.

Demonstrative pronouns (e.g., “*this*”*/*“*that*” in English) are among the most frequent words in the lexicons of the world's languages (Coventry & Diessel, 2025; Diessel & Coventry, 2020). It is generally accepted that they are used to create a joint focus of attention and to direct the attention of an addressee to an object or place (e.g., Burenhult, 2003; Clark, 1978; Clark & Sengul, 1978; Diessel, 2006; Talmy, 2020; Tomasello, 1999). It has also been established that a wide range of parameters affect demonstrative choice, including the distance between speaker and referent, relative positions of speaker and addressee, referent ownership, familiarity, visibility, elevation, and attention (e.g., see Diessel & Coventry, 2020; and Peeters, Krahmer, & Maes, 2021 for recent reviews). Yet, exactly how these factors are marshalled within specific languages is still much debated. Here, we explore two demonstrative systems that are usually assumed to operate very differently—Japanese and English—as a way of considering (and potentially reconciling) the variability of demonstrative theories within and between languages.

### 1.1 Theories of demonstratives: Egocentric versus person‐centered systems

**Fig. 1:**
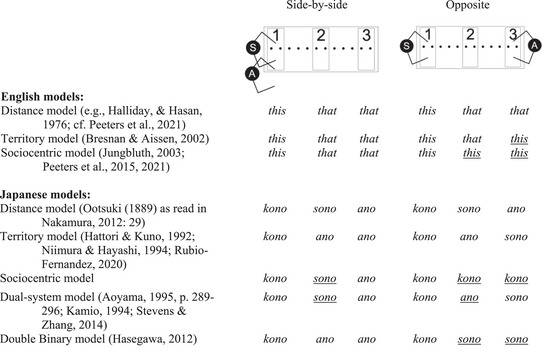
Overview of models of demonstrative use when referring to objects placed in each of the three regions in our experimental setup in Japanese and English, with an Addressee (A) seated either side‐by‐side or opposite the Speaker (S). As Japanese employs a three‐term system, we include two hybrid models that combine the distance and territory models in different ways. Underlined demonstratives mark the position differences in models.

One of the most common distinctions made with regard to demonstrative systems is whether a system is *person‐centered* or *non‐person‐centered/egocentric*. Around one‐quarter of the world's languages have person‐centered demonstrative systems that map onto the territories of both the speaker and/or the addressee (Breunesse, 2019). This is compared to the remainder of languages, which are generally thought to recognize only the egocentric space of the speaker. For example, while some accounts regarding how demonstratives operate in English have proposed that the relative positions of speaker and addressee are important for the choice between “*this*” and “*that*” (e.g., to disambiguate in which of the interlocutor's territories a referent is located; Bresnan & Aissen, 2002, see Fig. 1), the English demonstrative system is usually considered to be “non‐person‐centered” (Diessel, 1999; Levinson, 2018), with demonstratives indicating if a referent is close to (“*this*”) or further away from (“*that*”) a speaker (Anderson & Keenan, 1985; Diessel, 2013) (see Fig. 1).

In contrast to English, the Japanese (three‐term) demonstrative system is usually classified as person‐centered, with a dedicated term (“*sono*”) to mark object proximity with reference to a hearer (e.g., Aoyama, 1995, p. 289–296, see Fig. 1). While some early theorists treated Japanese demonstratives as marking the relative, graded, egocentric distance between a speaker and a referent (proximal demonstrative for nearby, medial term for further away, distal demonstrative for far away) (Diessel, 1999; Hasegawa, 2012; Niimura & Hayashi, 1994), more recent accounts have proposed that Japanese is a person‐centered system with a dedicated demonstrative marking that a referent is near an addressee (see, e.g., Aoyama, 1995, p. 289–296; Rubio‐Fernandez, 2020; Shin, Hinojosa‐Cantú, Shaffer, & Morford, 2020). Demonstratives, therefore, disambiguate which referent is talked about by specifying in which of the interlocutors’ *territories* the object is located.

An adaptation to the territory account is a *sociocentric* account, which proposes that when a speaker and an addressee are facing one another, the space between them is regarded as a uniform conversational territory. Referents within this space would be assigned the proximal demonstrative without further differentiation (e.g., Jungbluth, 2003; Peeters, Hagoort, & Özyürek, 2015, 2021; but see Rubio‐Fernandez, 2021). Yet, others have proposed hybrids of distance and territory accounts, where demonstratives are used differently depending on the relative positions of speaker and addressee. For example, the *dual system* account proposes that when speaker and addressee are side‐by‐side, demonstratives mark egocentric distance, but when the addressee is opposite, facing the speaker, demonstratives mark territory (Hoji, Kinsui, Takubo, & Ueyama, 2003; Stevens & Zhang, 2014). The last alternative is the *double binary* account, which makes two binary distinctions: when interlocutors are side‐by‐side, distance is contrasted (“*kono*”*/*“*ano*”), but when interlocutors face one another, “*kono*”*/*“*sono*” contrast territories. Although proposed accounts in the literature maintain that space is conceptualized in different ways in different situations (interlocutor configurations), mechanisms have not been provided to explain how different conceptualizations of space are established. The question, therefore, remains what the nature of addressee effects is, and whether they are specific to the languages with specific demonstrative terms that have been identified to mark addressee effects.

One might argue that further empirical research is needed to decide which of the many proposed accounts is the correct one for a given language. Alternatively, this theoretical diversity might simply reflect natural variation among speakers within a language. In a recent study, Coventry et al. (2023) tested speakers of 29 languages (Japanese and English among them) using the experimental manipulations shown in Fig. 1. They found that all tested languages have a term mapping onto reachable space (also called *peri‐personal space*, PPS from here) and another term mapping onto nonreachable space (or *extra‐personal space*, EPS from here), supporting earlier findings for the importance of reachability on demonstrative choice in English (Caldano & Coventry, 2019; Coventry, Valdés, Castillo, & Guijarro‐Fuentes, 2008), and consistent with a mapping between demonstratives and object manipulability (or simulation of an action) (Bufacchi & Iannetti, 2018; ter Horst, van Lier, & Steenbergen, 2011). Coventry et al. (2023) also provide evidence that the Japanese demonstrative system is among a cluster of languages showing addressee position effects, while the English system is not (results also supported for both Japanese and English by Rubio‐Fernandez, 2021, using an online task). However—and most relevant here—all 29 languages (including Japanese and English) showed significant variation in demonstrative use among speakers of individual languages, suggesting malleability within a language regarding how demonstratives can be used. Below, we explore the possibility that variability both between and within languages reflects the choice individual speakers might make regarding the *spatial perspective* to adopt—their own or the addressee's—potentially conjoining the mechanisms of demonstrative choice to those established for the so‐called *projective adpositions* (Carlson‐Radvansky & Logan, 1997).

### 1.2 Spatial demonstrative systems in perspective

**Fig. 2:**
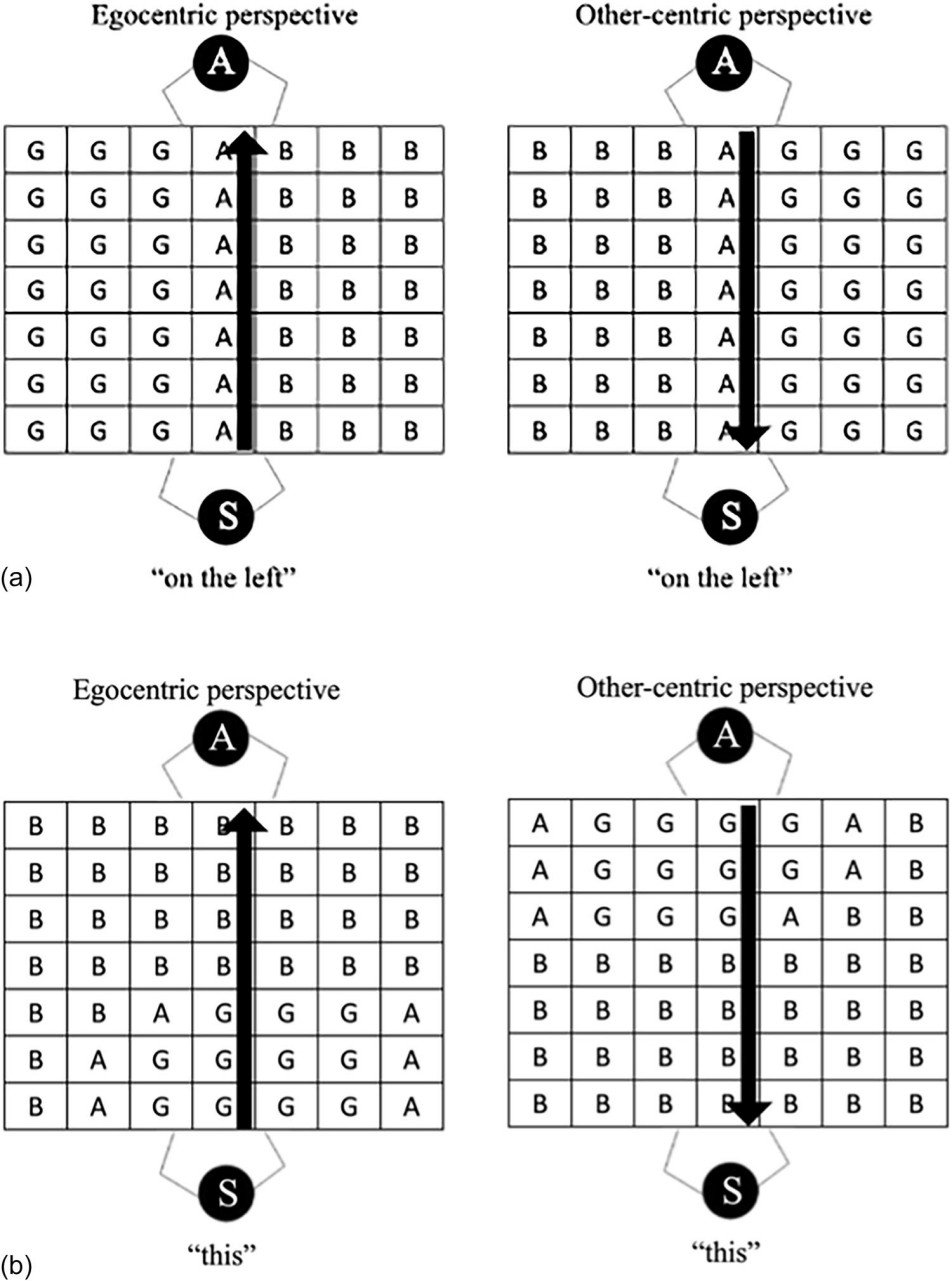
A speaker can describe space from their own egocentric perspective, or from the addressee's “other‐centric” perspective, changing the appropriateness of projective spatial adpositions (panel a). In panel b, a schematic representation of how “*this*” maps onto the world from an egocentric perspective (notice that the use is asymmetric, based on a speaker pointing with their right hand, based on Caldano & Coventry, 2019). “A,” “B,” and “G” in the grid refer to Acceptable, Bad, and Good regions for the use of the term.

In contrast to the normative view that languages employ an all‐or‐nothing system for demonstrative selection (the hypothesis implied in the demonstrative literature), we argue that demonstratives may operate like projective adpositions (terms such as “*to the left of*,” “*in front of*,” etc.), for which *spatial perspective taking* is important (Mainwaring, Tversky, Ohgishi, & Schiano, 2003; Schober, 1993, 1995; Schultheis, 2021; Tosi, Pickering, & Branigan, 2020; Tversky & Hard, 2009). In a series of language production studies, Tversky and Hard (2009) showed that participants can use either their own left‐right (egocentric) axis as a frame in which to use *“* *left of* *”* */* *“* *right of* *”*, or alternatively take the body axis of another person (see Fig. 2) (see also Tosi et al., 2020). For example, in one study, participants viewed photographs of a table with objects on it and a person seated on the opposite side, facing them. Participants sometimes spontaneously described the positions of the objects with reference to the person's left‐right axis rather than their own lateral axis. Moreover, in a condition where the person in the picture was reaching toward one of the objects, participants were twice as likely to take the other's perspective compared to a nonreach condition—even though there was obviously no communication with the person in the picture.

It has been suggested that speakers might switch perspectives to minimize the collective effort of interlocutors (Galati, Dale, & Duran, 2019; Galati & Avraamides, 2013). Moreover, taking into account how an addressee understands the world may implicate Theory of Mind (Rubio‐Fernandez & Jara‐Ettinger, 2020), with the speaker putting themselves in the addressee's shoes, potentially using a simulation of that agent's perspective and potential to act on an object as a basis for formulating their descriptions (ter Horst et al., 2011; Tosi et al., 2020). Outside of language, it has been shown that people automatically and effortlessly monitor someone else's perspective during perceptual decision‐making (Samson, Apperly, Braithwaite, Andrews, & Bodley Scott, 2010; Ward, Ganis, & Bach, 2019).

Extrapolating, we hypothesize that demonstratives can also be considered in terms of spatial perspective taking, with speakers choosing a perspective to adopt—either their own or the perspective of an addressee—in each communicative context. This leads to the consideration of a range of hypotheses as follows. First, we test if Japanese and English demonstrative systems should be treated as person‐centered and egocentric, respectively (in absolute terms), or alternatively if they are both malleable involving spatial perspective choice. To do so, we compare the use of demonstratives in a task with limited interaction between participants (secondary data from the Experiments in Coventry et al., 2023) with new Experiments where participants take part in the same task, but with increased interaction (i.e., comparing the case where only one person interacts with objects by placing them with the case where both participant and addressee interact with/place objects). If Japanese is person‐centered and English is egocentric, increased interaction should have no effect on the choice of demonstratives within each language to describe object location. In other words, if linguistic distinctions reflect absolute differences between speakers of different languages (the assumed view in linguistics) (e.g., Majid et al., 2004), then egocentric usage should be maintained even when the task context changes (we coin this the *absolute‐between‐language‐diversity hypothesis*). Such a hypothesis is consistent with the suggestion that individuals from interdependent cultures (e.g., East Asian cultures) have a more “interdependent” self, such that they are more likely to take another's perspective compared to individuals from more individualistic cultures (e.g., Western cultures) (Wu & Keysar, 2007). In contrast, if participants flexibly choose a spatial perspective to adopt, in line with approaches to spatial adpositions (i.e., consistent with the results from Tosi et al., 2020; Tversky & Hard, 2009), increased interaction (both participant and addressee act on/place objects) should lead to an increased likelihood of speakers taking the perspective of the addressee, with more person‐centered demonstrative usage across both languages (what we coin the *relative‐between‐language‐diversity hypothesis*). (Note that this is still compatible with Japanese speakers showing an overall higher likelihood of choosing the addressee‐perspective compared to English speakers.) Preliminary evidence for such a view comes from Rocca, Wallentin, Vesper, and Tylén (2019), who manipulated the degree of interaction/turn‐taking between (Danish) speakers and found evidence that proximal space/use of the proximal term is shifted toward the partner and remapped to the partner's reachable space, although they do not explore variation between participants or differences between languages.

To preview the results, we find evidence that increased interaction between speaker and addressee leads to an increase in person‐centered demonstrative use in both Japanese and English, consistent with the relative‐between‐language‐diversity hypothesis. Moreover, increasing interaction between participants shifts the *distributions of reference frame choices* within languages, again consistent with work on the use of spatial adpositions.

## 2 Method

We report the methods and results of the secondary data analyses and new Experiments together. Critically, the new Experiments used the same method as in the studies reported in Coventry et al. (2023), but with increased interaction between participants (i.e., participant and addressee taking turns to act on/place objects prior to spatial description).

2.1

#### 2.1.1 Participants

For the secondary data analyses, data were originally collected by Coventry et al. (2023)^1^ from 34 native Japanese^2^ speakers (age range: 19–54 [*M* = 27.35, *SD* = 7.43], 17 female) and 35 native English^3^ speakers (age range: 18–45 [*M* = 20.29, *SD* = 4.6], 18 female). For the new Experiments, data were collected from 33 Japanese speakers (age range: 19–49 [*M* = 26.5, *SD* = 8.25], 25 female) and 35 English speakers (age range: 18–67 [*M* = 24.83, *SD* = 11.32], 22 female). Participant numbers (a priori target of *N* = 32) were based on a power analysis (see Coventry et al., 2023). Stereoacuity was measured using the Randot Stereotest (Stereo Optical Inc., Chicago, USA), to ensure all participants had a depth perception of at least 40ʼʼ (arcseconds). Prior to analysis, data from eight participants were disregarded because they did not meet the depth perception threshold or did not engage with task instructions, and one participant chose to withdraw their data. All participants received course credit or a monetary reward for their participation.

#### 2.1.2 Procedure and design

All datasets (two secondary data analyses, two primary) were acquired using the “Spatial Memory Game paradigm” (Coventry et al., 2008; Gudde, Griffiths, & Coventry, 2018), in which participants had to remember the location of objects placed at different distances from them. Each participant was seated at a large conference table on which 12 locations were spaced along a midline from the participant's edge of the table, starting at 25 cm from the participant, up to 300 cm (see Fig. 1). The experimenter sat either side‐by‐side or opposite the participant, creating a binary manipulation of addressee position. On each trial, an object was placed in one of three conceptual regions (see Fig. 1): Region 1 (25 and 50 cm distance), where objects were within reach (PPS) of the speaker (participant), Region 2 (150 and 175 cm), at a medium distance from the speaker, and out of reach for both speaker and addressee (experimenter) in any spatial configuration, and Region 3 (275 and 300 cm), furthest from the speaker, but within the reachable space of the addressee, when seated opposite the participant. The difference between the original studies for which we ran a secondary analysis (Coventry et al., 2023) and our new Experiments is the amount of interaction that both parties (i.e., participant and addressee) had in the experiments. In the original studies, participants were seated, and all objects were placed by the experimenter. In our primary data studies, we doubled the number of trials, and both participant and addressee each placed the objects themselves for half of the trials. In line with previous studies (e.g., Rocca et al., 2019), we expected this increased interaction—with both participant and addressee acting on and placing objects throughout the experiments—to lead to more perspective taking.

Participants were told the study investigated the (possible) effects of language on memory for object location, and that they had been assigned to the *language condition*. This entailed that, following object placement, and after both interlocutors had returned to their seats, the participant (speaker) was asked to encode the object's location using both body and verbal language: point at (but not touch) the object and name it using a combination of three words: “[demonstrative (*kono/sono/ano; this/that*] [color] [shape],” for example, [“この赤い丸” (“*kono akai maru/this red circle*”)]. Participants were instructed that they could only use this three‐word structure so that every participant in the “language condition” experienced the same amount of verbal coding. They were encouraged to use all demonstratives across the trials, whatever felt natural to them (note that only one object was placed on each trial, so participants named a single object, avoiding contrastive referencing). Given that demonstrative use is very closely related to pointing behavior and eye‐gaze (Bangerter, 2004; Todisco, Guijarro‐Fuentes, Collier, & Coventry, 2020), we eliminated potential variation of language while pointing versus not pointing by asking participants to point at each object. In the Coventry et al. (2023) data, the object was always placed by the experimenter, but in the new Experiment, both participant and experimenter placed objects on an equal number of trials, as instructed by the experimenter at the start of each trial (e.g., “私/あなたは緑の点に赤い丸を配置します” (“*Watashi/Anata wa midori no ten ni akai maru wo haichi shimasu*”*)/*“*I/You place the red circle on the green dot*”), with the participant placing objects on half the trials.

The “language condition” cover was reinforced by asking participants for the most recent location of four of the previously placed objects on six different occasions throughout the study. This cover ensured participants did not guess the purpose of the study (confirmed upon debrief). All communication, from the moment participants entered the lab, occurred in the language of testing. The reanalysis data from Coventry et al. included 36 pseudo‐randomized (no object or distance was used in two successive trials) trials; 2 (position of the addressee) × 6 (distance), with three repetitions for each cell of the design. The new Experiments included an agency (2 (experimenter or participant places)) condition, doubling the total number of trials to 72. Data were analyzed based on the three conceptual Regions (near speaker, medium far/near addressee [only when positions were opposite], and far from both), so the six distances were combined in pairs.

#### 2.1.3 Statistical analyses

As each participant contributed multiple responses within the data set, the analyses needed to account for clustered or grouped data (an individual's multiple responses are likely to be correlated with each other). Multinomial multilevel modeling was chosen as the most appropriate means with which to analyze the data, as it allows the residual variance to be partitioned into a between‐participant component (the variance of the “clustered” participant‐level residuals) and a within‐participant component (the variance of the response‐level residuals) (Hoffman & Rovine, 2007; Sommet & Morselli, 2017). As there are only two response options in English (“*this*”/“*that*”), the English analysis is factually a binomial (rather than multinomial) analysis, but the analysis procedure is the same. All main effects and their interactions were included in the model. Data and analysis scripts are available online.^4^

While Coventry et al. (2023) focused on demonstrative use from the perspective of the speaker, contrasting Region 1 and the proximal demonstratives with other regions and demonstratives, our focus was to elicit any differences relevant for potential perspective taking. If demonstratives are used in a person‐centered fashion, addressee effects would be expected in Region 3, which is either close to the addressee, or far from both (or, in the case of the sociocentric hypothesis, the effect would also be in Region 2). Our analyses will elucidate whether speakers of Japanese and/or English produce demonstratives based on a fixed model (and if so, whether this is based on territory [person‐centeredness], distance, or a combination of the two), or whether participants produce demonstratives more flexibly, consistent with perspective taking.

### 2.2 Results and discussion

We first present a full model comprising all four Experiments (2 languages × 2 levels of interaction), followed by separate follow‐up models for the English and Japanese Experiments.

#### 2.2.1 Full model of all four Experiments (English and Japanese, original Experiments, and enhanced interaction Experiments)

The data are displayed in Table 1. To allow for comparison of all four Experiments in a single model, we recalculated the three‐term Japanese system to a binary distal (“*ano*”) versus nondistal (“*kono*” and “*sono*”) contrast to fit with the English two‐term system (“*that*” vs. “*this*”). A binomial multilevel model analysis was carried out with the following predictors: Position of Addressee (side‐by‐side, opposite), Region (3 levels), Language (English vs. Japanese), and Experiment (the original experiment from Coventry et al., 2023 vs. the Experiment with increased interaction), and all their interactions.^5^ The data were structured by individual responses clustered per participant. The reference outcome category was the distal demonstrative category. Categorical predictors were coded using effect coding (−0.5, 0.5) to facilitate interpretation of main effects as deviations from the grand mean, rather than differences from a single baseline category. Side‐by‐side (position), English, and Experiment 1 were coded as −0.5; and Opposite (position), Japanese, and Experiment 2 are 0.5. The three‐level factor Region was coded using Helmert contrasts, in which each level is compared to the mean of subsequent levels. The first contrast compared Region 1 to the other Regions (Region 1 = 0.5, Region 2 = −0.25, Region 3 = −0.25), the second contrast compared the second and third regions directly (Region 1 = 0, Region 2 = −0.5, Region 3 = 0.5). Using these contrasts ensures that Position effects are measured against the average across all regions, rather than depending on the reference region. The classification results can be found in Table 2.

**Table 1:** Frequencies and percentages of demonstrative use by region and addressee position for each Experiment

Experiment	Demonstrative	Position	Region 1	Region 2	Region 3
English original Experiment	this	Side‐by‐side	139 (72.40%)	33 (17.19%)	18 (9.38%)
		Opposite	125 (65.10%)	42 (21.88%)	19 (9.90%)
	that	Side‐by‐side	53 (27.60%)	159 (82.81%)	174 (90.62%)
		Opposite	67 (34.90%)	150 (78.12%)	173 (90.10%)
English increased interaction	this	Side‐by‐side	343 (89.32%)	90 (23.44%)	24 (6.25%)
		Opposite	334 (86.98%)	70 (18.23%)	41 (10.68%)
	that	Side‐by‐side	41 (10.68%)	294 (76.56%)	360 (93.75%)
		Opposite	50 (13.02%)	314 (81.77%)	343 (89.32%)
	kono	Side‐by‐side	192 (94.12%)	4 (1.96%)	3 (1.47%)
		Opposite	191 (93.63%)	4 (1.96%)	2 (0.98%)
Japanese original Experiment	sono	Side‐by‐side	9 (4.41%)	125 (61.27%)	22 (10.78%)
		Opposite	10 (4.90%)	140 (68.63%)	73 (35.78%)
	ano	Side‐by‐side	3 (1.47%)	75 (36.76%)	179 (87.75%)
		Opposite	3 (1.47%)	60 (29.41%)	129 (63.24%)
	kono	Side‐by‐side	364 (94.79%)	5 (1.30%)	2 (0.52%)
		Opposite	373 (97.14%)	12 (3.12%)	2 (0.52%)
Japanese increased interaction	sono	Side‐by‐side	20 (5.21%)	133 (34.64%)	33 (8.59%)
		Opposite	11 (2.86%)	216 (56.25%)	169 (44.01%)
	ano	Side‐by‐side	0 (0.00%)	246 (64.06%)	349 (90.89%)
		Opposite	0 (0.00%)	156 (40.62%)	213 (55.47%)

**Table 2:** Classification table for the MLM model, including all Experiments, overall percentage correct: 87.3%

		Observed	
		Nondistal	Distal
Predicted	Nondistal (*this/kono/sono*)	2896	392
		85.9%	10.9%
	Distal (*that/ano*)	497	3199
		14.6%	89.1%

**Fig. 3:**
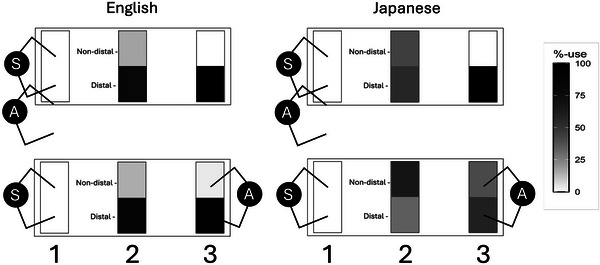
A grayscale visualization of the three‐way interaction Position by Region 2–3 contrast by Language superimposed on the tables as represented in Fig. 1. The use of distal and non‐distal demonstratives in grayscale represented within the Regions on the table, with side‐by‐side conditions in the top row, and opposite in the bottom row. Demonstratives are coded with nondistal demonstratives (“*this*,” “*kono*,” “*sono*”) in the top row within the Regions, and distal (*“* *that* *”*/*“* *ano* *”*) in the bottom row. While not as clear as the position effect in Japanese, in English, there is also a faint difference in the use of the nondistal demonstrative (“*this*”) with higher use in Region 3 in the opposite condition.

The first Region contrast, contrasting Region 1 with the other two Regions, was too strong a predictor and caused separation in the model, making the model results unreliable. Therefore, we removed that contrast. The final model shows a number of highly significant effects (Table 3), including main effects for Position, Region, and Language. When the odds ratio (OR) is >1, for example, for the main effect of Position, this suggests a higher likelihood (1.794 times likelier in this case) of a nondistal term when Position changes from side‐by‐side (coded as −0.5) to opposite (coded as 0.5), whereas the main effect in the Region 2‐Region 3 contrast (with an OR <1) suggests a higher likelihood of the reference category, suggesting the nondistal term is 0.193 times as likely as the distal term (or the distal term is 1/0.193 = 5.18 times more likely) when the Region changes from Region 2 to Region 3. The results of these main effects and interactions are consistent with the effects reported in Coventry et al. (2023). In this overall model, effects involving Experiment (original vs. increased interaction) and Language are of most interest. There is a strong Region by Experiment interaction, as well as a three‐way interaction between Position, the Region 2 by Region 3 contrast, and Language. This interaction is visualized in Fig. 3. The difference in the use of “*ano*” for Japanese participants in the contrast of Regions 2 and 3 is visible with higher use of “*sono*,” the perspective‐taking term, when the addressee is seated opposite (represented by a darker bar). The equivalent effect in English would be an increased use of “*this*,” which can be seen in Table 1. To understand how increased interaction changes addressee effects in each language, we follow‐up on this overall model with analyses per language. Moreover, given that “*kono*” and “*sono*” were collapsed in this overall model to fit the binary English system, further analysis is needed for Japanese to tease apart how the different Japanese terms are used across perspectives.

**Table 3:** Fixed effects of the overall model

	Estimate	SE	*z*	*p*	OR	CI_95
(Intercept)^**^	0.407	0.155	2.637	.0084	1.503	[1.11, 2.035]
Position^***^	0.585	0.158	3.704	.0002	1.794	[1.317, 2.445]
R2byR3 ^***^	−1.643	0.097	−17.015	< .001	0.193	[0.16, 0.234]
Region^***^	−4.509	0.189	−23.821	< .001	0.011	[0.008, 0.016]
Language^***^	2.594	0.31	8.381	< .001	13.386	[7.297, 24.554]
Experiment	0.125	0.281	0.445	.656	1.133	[0.654, 1.965]
Position×R2byR3^***^	0.897	0.191	4.695	< .001	2.452	[1.686, 3.565]
Position×Region^*^	0.919	0.362	2.541	.0111	2.507	[1.234, 5.093]
Position×Language^***^	1.202	0.316	3.808	.0001	3.326	[1.792, 6.173]
Position×Experiment	0.445	0.315	1.413	.1577	1.561	[0.842, 2.895]
R2byR3×Language^***^	−1.064	0.189	−5.638	< .001	0.345	[0.238, 0.5]
Region×Language^***^	−3.026	0.378	−8.011	< .001	0.048	[0.023, 0.102]
R2byR3×Experiment	0.317	0.194	1.634	.1022	1.373	[0.939, 2.008]
Region×Experiment^***^	−1.16	0.186	−6.22	< .001	0.314	[0.218, 0.452]
Language×Experiment	−0.786	0.565	−1.391	.1643	0.456	[0.151, 1.379]
Position×R2byR3×Language^**^	1.076	0.382	2.815	.0049	2.932	[1.387, 6.199]
Position×Region×Language	1.066	0.723	1.473	.1407	2.902	[0.703, 11.98]
Position×R2byR3×Experiment	0.707	0.381	1.856	.0634	2.028	[0.961, 4.279]
Position×Region×Experiment	0.443	0.723	0.613	.5401	1.557	[0.377, 6.425]
Position×Language×Experiment	0.895	0.629	1.422	.1551	2.447	[0.713, 8.403]
Position×R2byR3×Language×Experiment	−0.908	0.756	−1.201	.2299	0.403	[0.092, 1.776]
Position×Region×Language×Experiment	1.301	1.445	0.9	.3682	3.672	[0.216, 62.41]

Significance codes: <.001 “^***^”; <.01 “^**^”; <.05 “^*^”.

The within‐participant random effect was significant (*ICC* = 0.411), consistent with the findings of Coventry et al., 2023; indicating that 41.1% of the variance is accounted for by the clustering of responses at the individual level. We will discuss this further in the exploratory Section 2.2.2.

##### 2.2.1.1 Unpacking the three‐way interaction, comparing the original Experiment with the Experiment with increased interaction in English

The English demonstrative data are displayed in Table 1. A binomial multilevel model analysis was carried out with the following predictors: Position, Region, and Experiment. The data are structured by individual responses clustered per participant. The reference outcome category was the distal demonstrative category. Categorical predictors were coded using effect coding (−0.5, 0.5) to facilitate interpretation of main effects as deviations from the grand mean, rather than differences from a single baseline category. Side‐by‐side (position), and the original Experiment were coded as −0.5; and Opposite (position), and the Experiment with increased interaction are 0.5. The three‐level factor Region was coded using Helmert contrasts, in which each level is compared to the mean of subsequent levels. The first contrast compared Region 1 to the other Regions (Region 1 = 0.5, Region 2 = −0.25, Region 3 = −0.25), the second contrast compared the second and third regions directly (Region 1 = 0, Region 2 = −0.5, Region 3 = 0.5). As “*that*” is the baseline, a significant effect with an OR >1 suggests that, based on the experiment's data, there is a higher likelihood of “*this*” compared to “*that*.” The model's classification results can be found in Table 4.

**Table 4:** Classification table for the binomial multilevel English model, overall percentage correct: 85.3%

		Observed	
		Proximal	Distal
Predicted	Proximal (*this*)	946	176
		74%	8.1%
	Distal (*that*)	332	2002
		26%	91.1%

**Fig. 4:**
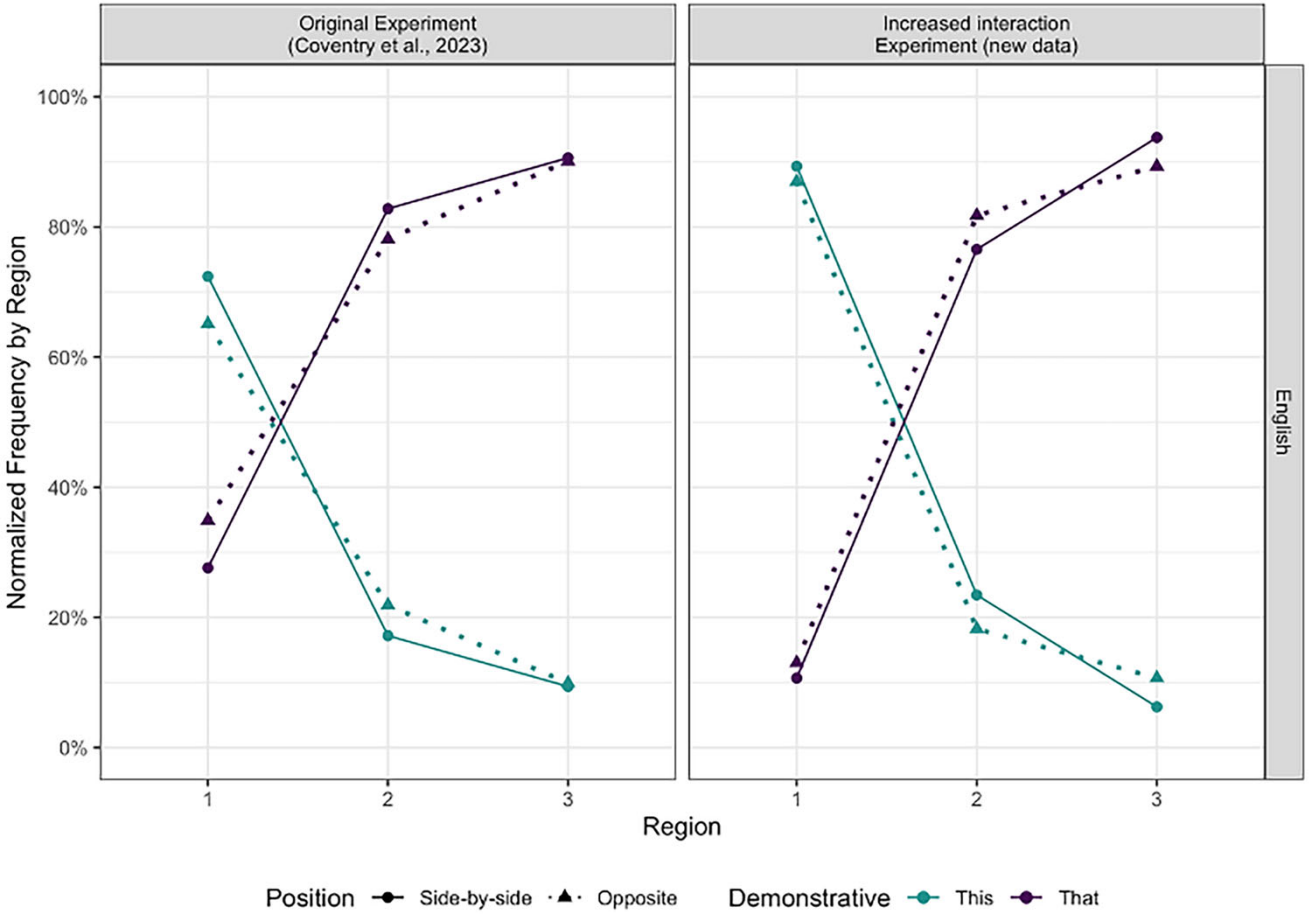
English demonstrative use by Region and Position in both the Original experiment and the Experiment with increased interaction.

The model shows a significant main effect of Region. The OR of 0.055 suggests that “*this*” is less likely to be a response (or, in other words, that “*that*” is 1/0.055 = 18.18 times more likely), as the location of the object changes to further regions. The significant interaction of Position by the Region 2‐Region 3 contrast shows that this is not just in Region 1, but also between Regions 2 and 3. There was also an interaction between Position and Region, but this interaction is also mediated by Experiment, given the significant Position by Region by Experiment interaction. “*This*” is used more often in Region 1 in the increased interaction Experiment. This could be a function of different participants between the two experiments, or the increased interaction in the new Experiment emphasizes the marking of one's own space. Furthermore, there is a crossover between Region 2 and Region 3, where “*this*” is used less frequently in Region 2 in the increased interaction Experiment when the addressee is seated opposite, but more in Region 3, with an OR indicating a 3.362 times higher likelihood. This effect is not there in the original experiment (Table 5, visualized in Fig. 4). These effects suggest that English speakers are sensitive to the location of a conspecific. While the default is to produce demonstratives from an egocentric perspective in English, when speakers produced their demonstratives in a more collaborative setting, they took the addressee's perspective more often when the addressee was seated opposite the speaker and the object was located within the addressee's PPS.

**Table 5:** Fixed effects of the model across English Experiments

	Estimate	SE	*z*	*p*	OR	CI_95
(Intercept)^***^	−0.845	0.117	−7.219	< .001	0.429	[0.341, 0.54]
Position	0.003	0.107	0.026	.9791	1.003	[0.813, 1.237]
R2byR3^***^	−0.999	0.138	−7.232	< .001	0.368	[0.281, 0.483]
Region^***^	−2.904	0.093	−31.366	< .001	0.055	[0.046, 0.066]
Experiment	0.413	0.234	1.763	.0779	1.511	[0.955, 2.392]
Position×R2byR3	0.351	0.276	1.274	.2028	1.421	[0.828, 2.44]
Position×Region^*^	0.387	0.176	2.205	.0275	1.473	[1.044, 2.078]
Position×Experiment	0.014	0.214	0.067	.9469	1.014	[0.667, 1.544]
R2byR3×Experiment	−0.295	0.276	−1.068	.2857	0.745	[0.433, 1.28]
Region×Experiment^***^	−1.106	0.185	−5.975	< .001	0.331	[0.23, 0.475]
Position×R2byR3×Experiment^*^	1.212	0.552	2.198	.0279	3.362	[1.14, 9.91]
Position×Region×Experiment	−0.173	0.351	−0.493	.6218	0.841	[0.422, 1.674]

Significance codes: <.001 “^***^”; <.01 “^**^”; <.05 “^*^”.

##### 2.2.1.2 Unpacking the three‐way interaction, comparing the original Experiment with the Experiment with increased interaction in Japanese

As can be seen in the frequency data in Table 1, there was no variation between Position conditions in the low number of “*ano*” responses in Region 1. This caused a separation in the model. As the interesting effects are hypothesized to occur between “*ano*” and “*sono*” in Regions 2 and 3, we removed Region 1 from the analysis, keeping the rest of the model the same. A multinomial multilevel model analysis was carried out with the following predictors: Position, Region, and Experiment. The data were structured by individual responses clustered per participant.

The reference outcome category was the distal demonstrative category. Categorical predictors were coded using effect coding (−0.5, 0.5) to facilitate interpretation of main effects as deviations from the grand mean, rather than differences from a single baseline category. Side‐by‐side (position), and the original Experiment were coded as −0.5; and Opposite (position), and the Experiment with increased interaction are 0.5. As Region 1 is taken out, Region is also a binary contrast now with Region 2 = −0.5, Region 3 = 0.5.

“*Sono*” is chosen as the reference category, as it occurs in both contrasts in demonstrative production. Therefore, there is an estimate for the *sono*‐*kono* or *sono*‐*ano* contrast in each condition.

**Fig. 5:**
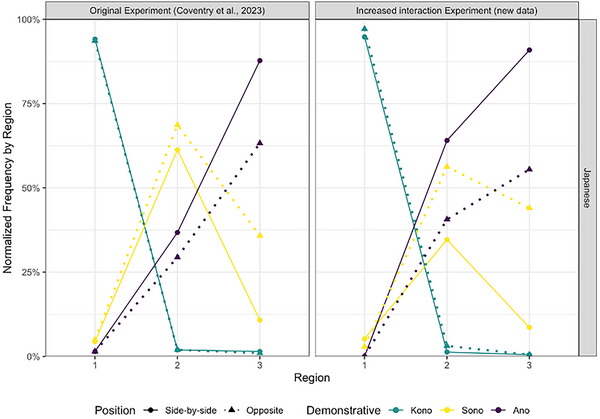
Japanese demonstrative use by Region and Position in both the Original experiment and the Experiment with increased interaction.

As Region 1, the region where “*kono*” is used almost exclusively, was not included in the model, the classification table (Table 6) does not predict any proximal demonstrative use. Furthermore, the low number of “*kono*” responses remaining in the data after removing Region 1 also leads to no effects being found with “*kono*.” The fixed effects table (Table 7) shows main effects of Position, Region, and Experiment in the *sono‐ano* contrast. Furthermore, as the log odds ratios in the two‐way interactions with each combination of Position, Region 3, and Experiment are OR<1, this shows increased odds for the reference condition (“*sono*” compared to “*ano*” in these cases). The three interactions with “*ano*” show that: (1) in our data, speakers are (1/0.2 =) 5 times more likely to use “*sono*” when the object is located in Region 3, and the addressee is seated opposite, compared to the addressee sitting side‐by‐side (the Position by Region 3 contrast). (2) The likelihood of a “*sono*” response is (1/0.299 =) 3.44 times higher based on a position change in the Experiment with increased interaction compared to the original Experiment (the Position by Experiment contrast), and (3) that a “*sono*” response is (1/0.363 =) 2.7 times more likely for an object in Region 3 in Experiment 2 compared to Experiment 1. This suggests that Japanese demonstrative choice is affected by the position of the addressee, but also that this effect is stronger in the version of the Experiment where there is more interaction of the participant during the experiment. Fig. 5 visualizes these effects. In the original Experiment, “*sono*” was the demonstrative of choice for Region 2, but was used as a medial term, with less than 10% use in both Regions 1 and 3. That is replicated in the side‐by‐side condition in the experiment with increased interaction. However, when the addressee is seated opposite, participants’ demonstrative choice changed, consistent with the idea that “*sono*” might have two functions: it is a middle term, but also signals perspective taking, where “*sono*” is used to indicate objects close to the addressee. When there is more interaction, speakers are more likely to take another's perspective and use “*sono*” in a person‐centered way, compared to a medial‐distance term.

**Table 6:** Classification table for the MLM of the Japanese experiments, overall percentage correct: 83.8%

		Observed		
		Proximal	Medial	Distal
Predicted	Proximal (*kono*)	0	0	0
		0%	0%	0%
	Medial/Addressee	13	740	117
	term (*sono*)	38.2%	81.2%	12.6%
	Distal (*ano*)	21	171	1230
		61.8%	18.8%	87.4%

**Table 7:** Fixed effects of the model across Japanese experiments

	Estimate	SE	*z*	*p*	OR	CI_95
kono∼(Intercept)^***^	−3.045	0.317	−9.607	< .001	0.048	[0.026, 0.089]
ano∼(Intercept)^**^	0.961	0.308	3.12	.0018	2.615	[1.43, 4.782]
kono∼Position^**^	−1.176	0.43	−2.738	.0062	0.308	[0.133, 0.716]
ano∼Position^***^	−1.958	0.144	−13.604	< .001	0.141	[0.107, 0.187]
kono∼R2byR3	0.596	0.432	1.381	.1673	1.815	[0.779, 4.23]
ano∼R2byR3^***^	2.41	0.147	16.389	< .001	11.137	[8.348, 14.857]
kono∼Experiment	0.342	0.634	0.54	.589	1.408	[0.407, 4.879]
ano∼Experiment	0.907	0.616	1.472	.141	2.477	[0.74, 8.285]
kono∼Position×R2byR3^*^	−2.161	0.855	−2.527	.0115	0.115	[0.022, 0.616]
ano∼Position×R2byR3^***^	−1.611	0.281	−5.73	< .001	0.2	[0.115, 0.347]
kono∼Position×Experiment	−0.011	0.859	−0.012	.99	0.989	[0.184, 5.329]
ano∼Position×Experiment^***^	−1.207	0.288	−4.193	< .001	0.299	[0.17, 0.526]
kono∼R2byR3×Experiment	−1.445	0.863	−1.674	.0942	0.236	[0.043, 1.28]
ano∼R2byR3×Experiment^***^	−1.012	0.294	−3.441	.0006	0.363	[0.204, 0.647]
kono∼Position×R2byR3×Experiment	−0.579	1.71	−0.339	.7347	0.56	[0.02, 15.991]
ano∼Position×R2byR3×Experiment	0.044	0.562	0.079	.937	1.045	[0.347, 3.147]

*Note*. The demonstrative in each row shows whether the effect represents the contrast between “*sono*” and “*kono*,” or “*sono*” and “*ano*.”

Significance codes: <.001 “^***^”; <.01 “^**^”; <.05 “^*^”.

The within‐participant random effect was again significant (*ICC* = 0.44); indicating that 44% of the variance is accounted for by the clustering of responses at the individual level.

Importantly, the same three‐way interaction comes out in both languages, with the effect going in the same direction: in the Experiment with increased interaction, there is a higher likelihood of the terms that signal *perspective taking (*“*this*,” “*sono*”) when the object is placed within the PPS of the addressee. While the effect of position has a higher weighting in Japanese, English participants in the Experiment with increased interaction showed sensitivity to the addressee's position as well.

In all three models, we find significant within‐participant, random effects, suggesting that clustering by participants accounts for part of the variation. As we are reluctant to a posteriori divide participants into artificial groups, we cannot perform any confirmatory analysis on these random effects. Furthermore, models to statistically test differences between the original Experiments versus the enhanced interaction Experiments in Japanese or English do not converge because selecting trials in Region 3 in which the addressee is seated opposite leaves too few trials for appropriate statistical power (cf. Brysbaert & Stevens, 2018). This leaves us with a descriptive exploration of the data in the next section.

#### 2.2.2 Additional exploration

**Fig. 6:**
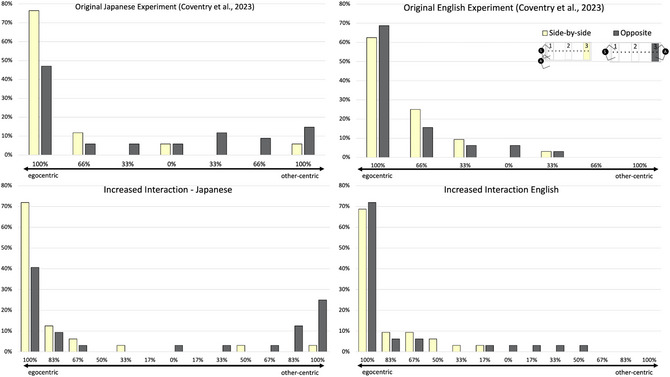
These graphs represent the percentage of participants responding from an egocentric or other‐centric reference frame when the object was placed in Region 3 for each addressee position. The x‐axis represents the percentage bias toward either perspective (on a scale of full egocentric to full other‐centric, 0% means equal use of both perspectives). In the Coventry et al. dataset (top row), there were six trials in this cell of the design; in the new datasets (bottom row), 12. The y‐axis shows the percentage of participants, for example, in the original Japanese Experiment, 26 of 34 (76%) participants always using “*ano*,” and thus the egocentric perspective in side‐by‐side trials, 5/34 (15%) participants always chose “*sono*,” and thus the other‐centric perspective, when the addressee was seated opposite.

Rather than speakers of both languages adhering to a language‐specific “fixed” demonstrative system, the data are consistent with the view that speakers use demonstratives much more flexibly. Further, exploratory interrogation of the data (see Fig. 6) suggests the nature of this variation in terms of differences between Japanese speakers in their use of “*sono*.” Only 5 out of 34 participants always used “*sono*” when the object was close to the addressee (i.e., using “*sono*” in a person‐centered fashion), compared to 16 participants who never used “*sono*,” and thus arguably chose an egocentric reference frame. The rest of the participants (around 40%) switched between “*sono*” and “*ano*” in these trials, suggesting that speakers use demonstratives flexibly, even when circumstances such as relative distance between speaker and referent, and the spatial configuration of interlocutors are identical. So, rather than being *obligatory* to produce or to comprehend “*sono*” from the perspective of the speaker/addressee, the results are likely to be dependent on participants’ choice of perspective, which will naturally vary from experiment to experiment and trial to trial (consistent with work on spatial adpositions). Moreover, in the more interactive Experiment, there is an overall shift in the distribution toward other‐centric use, with 25% of participants using “*sono*” in all trials in a person‐centered fashion in Region 3 compared to 15% in the Coventry et al. data.

In the analysis of the English data, we found little evidence of perspective taking when participants freely produced demonstratives to describe object locations in the original Experiment (see Fig. 6, or Table 1); 22 of the 32 participants never used “*this*” when the object was in the PPS of the addressee when the addressee was seated opposite, and no participant always used “*this*.” However, if demonstrative production might be subject to perspective taking like other spatial language, one would expect a shift toward perspective taking when interaction is increased (Rocca et al., 2019; Tosi et al., 2020; Tversky & Hard, 2009). While in the original Experiment, only one participant used “*this*” more often than “*that*” in trials in which the object was in Region 3, with the addressee seated opposite (thus leaning toward the other‐centric use), three English speakers did so in the increased interaction Experiment.

In both languages, we observe that when both participant and addressee take turns to place the object on each trial, hence both acting upon the object on different trials, this elicits a more frequent choice of the other‐centric frame. While English does not have a demonstrative dedicated to communicating person‐centered information, English speakers are nevertheless sensitive to the position of the addressee, with a small but significant increase in the use of “*this*” to refer to objects located in the PPS of an addressee when the object is in the EPS of the participant.

## 3 General discussion

Our main goal was to examine the nature of supposed differences between so‐called “*person‐centered*” and “*non‐person‐centered/egocentric*” demonstrative systems, focusing on Japanese and English demonstrative systems as exemplars of each type of system. Specifically, our aim was to test between the view that demonstrative systems in languages are either person‐centered or egocentric (the *absolute‐between‐language‐diversity hypothesis*) or alternatively that demonstrative systems can be used flexibly across languages, with languages exhibiting different (but malleable) preferences for perspective taking (the *relative‐between‐language‐diversity hypothesis*). We varied the extent to which speaker and addressee were both involved in placing objects prior to description (hence manipulating the interaction between participants) during the Experiments to test between these alternative views. As we discuss below, the results favor the latter hypothesis.

To begin with, our results tease apart five different accounts of the Japanese demonstrative system, varying from a treatment of Japanese demonstratives as purely egocentric (e.g., Nakamura, 2012), to accounts proposing that Japanese demonstratives can signify various contrasts, in which “*sono*” is described to have different meanings: egocentric, person‐centered, or a combination of the two (Hasegawa, 2012; Hattori & Kuno, 1992; Nakamura, 2012). The data show across Experiments that “*kono*” is used almost exclusively in egocentric reachable space (PPS), “*ano*” is used to describe object locations out of egocentric reach (EPS), and overall “*sono*” appears to have a dual function of serving as a middle‐distance term and describing a referent in the addressee's PPS. Therefore, the results are most in line with the dual‐system model, in which “*sono*” can be used as a medial distance term, or a person‐centered term indicating a referent is near the addressee. However, the use of “*sono*” in our data is more flexible than described in the literature. The meaning of “*sono*” is often described as a function of the spatial configuration of interlocutors (e.g., when speaker and addressee have a shared perspective—i.e., are side‐by‐side—“*sono*” is a medial term; when interlocutors have opposite perspectives, “*sono*” is suggested to be a person‐centered term) (Aoyama, 1995; Kamio, 1994; Stevens & Zhang, 2014). In our Experiments, participants seem to switch rather flexibly between these functions, influenced, but not determined, by the environment.

Similarly, there are different accounts of the English demonstrative system, sometimes described as completely egocentric (e.g., Halliday, & Hasan, 1976), other times with recognized potential for incorporating addressee positions, either in the form of territory effects (e.g., Bresnan & Aissen, 2002) or shared space (e.g., Jungbluth, 2003; Peeters et al., 2015, 2021). On the face of it, our data are most in line with the view that English demonstratives are used egocentrically, with “*this*” used then the object being referred to is in the PPS of the speaker and “*that*” when the object is outside of PPS (consistent with previous results: see Coventry et al., 2023; Coventry & Diessel, 2025).

On the surface, Japanese seems person‐centered, while English seems non‐person centered. However, across the two experimental designs varying in the extent to which participants interact, our data reveal a shift in the use of demonstratives in *both* languages. This is consistent with the view that speakers in both languages use demonstratives flexibly, and shift toward an increased likelihood of adopting the addressee's (other‐centric) perspective when a subtle manipulation increases the interaction between speaker and addressee in the Experiment. Specifically, we found the same significant three‐way interaction between Experiment, Position, and Region for both languages, with an increased use of “*sono*” and “*this*” in the PPS of the addressee in the more interactive Experiment. This is consistent with work on spatial adpositions that have shown that increase interaction led to an increased tendency to describe an object as to left or right of an addressee (i.e., with respect to the addressee's left/right axis; Tversky & Hard, 2009; Tosi et al., 2020).

In both languages, we also found significant variation in how speakers within a language use demonstratives, consistent with the findings across 29 languages reported by Coventry et al. (2023). Moreover, the experimental designs, varying the extent to which participants interact, reveal a nuanced pattern of demonstrative use across languages, with some speakers shifting perspective, while others do not. Importantly, the variation across individual participant's trials indicates that a speaker can still choose to mark egocentric distance when the speaker and addressee are facing one another. Previous literature treats the Japanese demonstrative system as all or none, in which demonstrative use is fixed dependent on conversational setting, rather than the speaker flexibility we find in our data. The choice of perspective can still be biased by the weight of specific parameters (be it language, conversational setting, or the interplay of cognitive mechanisms) (Dale et al., 2018), but the fact that speakers need to choose will lead to within‐language differences.

We echo past acknowledgments that demonstrative choice is affected by multiple parameters (Coventry, Griffiths, & Hamilton, 2014; Diessel & Coventry, 2020; Peeters et al., 2021), but critically introduce a perspective taking account of demonstrative use (in line with work on perspective taking in general, see, e.g., Galati et al., 2019). As has long been established in other types of cognition as well as spatial language, speakers can choose to take an ego‐ or other‐centric perspective. The optionality of perspective taking may also help explain the diversity of theoretical approaches to Japanese demonstratives, with linguists often basing their accounts on small numbers of informants, hence potentially misattributing optional parameters for those assumed to be obligatory—especially given the between‐participant variation we observed in our data. While our Japanese data most closely align with a dual‐system account (Niimura & Hayashi, 1994; Okazaki, 2011; Takahashi, 1992), in which speakers can choose to use demonstratives to encode distance and person‐centeredness, speakers can opt to use demonstratives egocentrically even when the addressee is positioned opposite the speaker.

Demonstrative production is a function of interacting parameters (e.g., physical, psychological, cf. Peeters et al., 2021), informing how demonstratives contrast parameters in given settings. Reduction in interaction may reduce the chances of finding perspective taking, consistent with recent findings indicating that the degree of interaction can affect how spatial demonstratives are used (Rocca et al., 2019). Approaching diversity through consideration of both between‐ and within‐language variation is essential to unpack the nature of linguistic diversity, with various parameters affecting language production in a probabilistic model.

Overall, our findings suggest that speakers of *both* languages can take the position of an addressee into account, although the Japanese demonstrative system affords a more systematic and explicit means to do so, with a dedicated demonstrative to denote whether an object is reachable by the addressee. Hence, these results are consistent with the view that languages may vary in the explicit contrasts their demonstrative systems possess (see, e.g., Levinson, Cutfield, Dunn, Enfield, & Meira, 2018; Diessel, 1999, 2005), but speakers of languages lacking terms for specific contrasts may nevertheless use the demonstratives available in their language to make distinctions as a function of the same underlying parameters (Coventry et al., 2014).

### 3.1 Spatial demonstratives and perspective taking: Toward a new hypothesis of demonstrative choice

Our results implicate that speakers can *choose* to take their own perspective or an other‐centric perspective. It has usually been assumed that spatial demonstratives do not operate within spatial frames of reference (Garnham, 1989; Levinson, 1996; Logan & Sadler, 1996). For example, Garnham (1989) notes that demonstratives are associated with basic relations that take only one argument that expresses the distance of an object with respect to the viewer. Such single argument relations are taken to map onto crude representations of space without the need for more precise specification about the objects or locations involved, in contrast to adpositions that typically involve a viewer, a located, and a reference object. We suggest that demonstratives should not be considered so crudely and that the same underlying processes may apply across a wide range of spatial terms not usually considered together. For example, speakers first need to know whether the object's location is to be described from the perspective of the speaker or the addressee (Samson et al., 2010). This could be construed as selecting a reference object and/or selecting a reference frame, dependent on definitions/taxonomies adopted. Moreover, Levinson and colleagues (2018) suggest that available reference frames may include the absolute frame, given evidence that the demonstrative systems of some languages encode absolute direction (Burenhult, 2018; Van Staden, 2018).

Importing a framework proposed for spatial adpositions, this process can be conceptualized in a series of steps that involve first (1) selecting a reference object, (2) selecting a reference frame from available reference frames, (3) superimposing the origin of the reference frame on the reference object, (4) orienting the axes with respect to the defining source of information, (5) identifying the direction assigned to the spatial relation by the relevant axis of the reference frame, and then (6) checking this relation against spatial templates for different relations to find the best description to match the perceptual relation (Carlson & Van Deman, 2004; Carlson‐Radvansky & Logan, 1997; Logan & Sadler, 1996).

We hypothesize that many of the so‐called “social” factors that have been marshalled as an argument against the mapping between spatial demonstratives and perceptual space (see, e.g., Peeters & Özyürek, 2016) may well be accommodated by the likelihood with which a speaker chooses to describe the world from another's perspective. Moreover, there is evidence that the processing of space itself is affected by (seating) positions and the degree of interaction between participants during a task (Lukošiūnaitė, Kovács, & Sebanz, 2024; Rocca et al., 2019; Rubio‐Fernandez, 2022; Teneggi, Canzoneri, di Pellegrino, & Serino, 2013). For example, Teneggi and colleagues show that PPS shrinks when people are facing each other, and having a conspecific acting within one's PPS affects (cross‐modal) processing of PPS (Heed, Habets, Sebanz, & Knoblich, 2010; Pellencin, Paladino, Herbelin, & Serino, 2018). Given that participants have to mechanistically process the distances of objects as well as addressee positions to inform their language choices to communicate about space, understanding the processing of space as well as addressee positions will benefit from more of a neurocomputational model. Consistent with this, studies have shown a subpopulation of place cells in the dorsal hippocampus of bats and rats that specifically represent the position of a conspecific in allocentric coordinates, suggesting that spatial representations include both self and nonself (Danjo, Toyoizumi, & Fujisawa, 2018; Omer, Maimon, Las, & Ulanovsky, 2018).

The presented data provide further evidence for the view that differences in demonstrative systems between languages (Diessel, 2006; Levinson et al., 2018) may represent variability in the explicitness with which demonstrative systems recognize parameters that are nevertheless reflected in frequencies of demonstrative use more universally (Bresnan & Aissen, 2002; Coventry et al., 2014; Johannes, Wilson, & Landau, 2016). Such a view runs contrary to the claims made by Evans and Levinson (Evans & Levinson, 2009) that spatial communication systems are fundamentally different across languages. Testing a range of variables across a diverse range of demonstrative systems is the next natural step to definitively test the universal hypothesis, a line of work we are currently undertaking.

## Conflict of interest

None of the authors has a conflict of interest to declare.

### Ethics statement

Ethical approval was granted by the University of East Anglia School of Psychology Ethics Committee.

## Supporting information



Supplementary Information

## Data Availability

All data are available via links in footnotes in the main manuscript.

## Notes

1 The dataset is available at https://osf.io/ush2w/.

2 Japanese speakers were (temporarily) living in the UK (most were attending a UK university or foundation course, with teaching in English), but had learned Japanese from birth and completed primary and high school education in Japanese. Therefore, all participants were bilingual, but dominant (L1) in Japanese.

3 All were native and functionally monolingual English speakers. Some participants reported high‐school taught knowledge of other languages, but would not be able to converse in these languages.

4 All data and script available here: https://osf.io/r8ehv/?view_only=d8a949ea13574ffa92880055543fd21f.

5 We ran an analysis also including who placed the object, but this model did not converge. For transparency, we report an additional analysis across the two relevant experiments with “who placed” as predictor in the Supplementary Materials. This analysis revealed an independent interaction between language and who placed the object, but no other interactions that affect the interpretation of the findings presented in the main manuscript.
