# Supplementary material for: Spatial Demonstratives and Perspective Taking in English and Japanese
Source: Cogn Sci. 2026 Mar 23;50(3):e70183. doi: 10.1111/cogs.70183 (PMC13007274; doi:10.1111/cogs.70183)
Supplement: Supplementary file 1 — Supplementary Information [file COGS-50-e70183-s001.docx]

## Supplementary Materials

To explore whether the *who places* effect (whether the Experimenter or the Participant placed the object for a given trial) affected demonstrative use, we ran an additional analysis to include who places as predictor in the analysis comparing the experiments with increased interaction, between languages.

The data for both languages are displayed in Table S1. A binomial multilevel model analysis was carried out with the following predictors: Position, Region, Language, and Agent. The data is structured by individual responses clustered per participant. The reference outcome category was the distal demonstrative category, in contrast to non-distal responses (*this* in English, *kono/sono* in Japanese).

| Table S1. Frequencies and percentages of demonstrative use by region, addressee, and agent for both languages | | | | | | |
| --- | --- | --- | --- | --- | --- | --- |
| **Experiment** | **Demonstrative** | **Position** | **Who places** | **Region_1** | **Region_2** | **Region_3** |
| English increased interaction | NonDistal | Side-by-side | Experimenter | 169 (88.02%) | 42 (21.88%) | 8 (4.17%) |
|  | Distal | Side-by-side | Experimenter | 23 (11.98%) | 150 (78.12%) | 184 (95.83%) |
|  | NonDistal | Opposite | Experimenter | 158 (82.29%) | 30 (15.62%) | 18 (9.38%) |
|  | Distal | Opposite | Experimenter | 34 (17.71%) | 162 (84.38%) | 174 (90.62%) |
|  | NonDistal | Side-by-side | Participant | 174 (90.62%) | 48 (25.00%) | 16 (8.33%) |
|  | Distal | Side-by-side | Participant | 18 (9.38%) | 144 (75.00%) | 176 (91.67%) |
|  | NonDistal | Opposite | Participant | 176 (91.67%) | 40 (20.83%) | 23 (11.98%) |
|  | Distal | Opposite | Participant | 16 (8.33%) | 152 (79.17%) | 169 (88.02%) |
| Japanese increased interaction | NonDistal | Side-by-side | Experimenter | 12 (100.00%) | 69 (36.32%) | 20 (10.42%) |
|  | Distal | Side-by-side | Experimenter | 0 (0.00%) | 121 (63.68%) | 172 (89.58%) |
|  | NonDistal | Opposite | Experimenter | 7 (100.00%) | 113 (59.79%) | 85 (44.27%) |
|  | Distal | Opposite | Experimenter | 0 (0.00%) | 76 (40.21%) | 107 (55.73%) |
|  | NonDistal | Side-by-side | Participant | 8 (100.00%) | 64 (33.86%) | 13 (6.84%) |
|  | Distal | Side-by-side | Participant | 0 (0.00%) | 125 (66.14%) | 177 (93.16%) |
|  | NonDistal | Opposite | Participant | 4 (100.00%) | 103 (56.28%) | 84 (44.21%) |
|  | Distal | Opposite | Participant | 0 (0.00%) | 80 (43.72%) | 106 (55.79%) |

Just like in the analyses in the main manuscript, the lack of variance in Region 1 causes separation, so in the actual model we excluded Region 1. Categorical predictors were coded using effect coding (-0.5, 0.5) to facilitate interpretations of main effects as deviations from the grand mean, rather than differences from a single baseline category. The following conditions were coded as -0.5: English experiment, Region 2, Side-by-side (position), Participant places. Demonstrative responses were coded as distal (*that/ano)* vs nondistal (*this/kono/sono)*. The Classification table can be found in Table S2.

| Table S2. Classification table for the MLM model including all experiments, overall percentage correct: 85.2% | | | |
| --- | --- | --- | --- |
| Predicted |  | Observed | |
|  |  | Non-distal | Distal |
|  | Non-distal (*this/kono/sono*) | 511 | 187 |
|  |  | 65.9% | 8.2% |
|  | Distal (*that/ ano*) | 265 | 2088 |
|  |  | 34.1% | 91.8% |

| Table S3. Fixed effects of the overall model. | | | | | | |
| --- | --- | --- | --- | --- | --- | --- |
|  | Estimate | SE | z | p | OR | CI_95 |
| (Intercept)*** | -2.074 | 0.304 | -6.832 | <0.001 | 0.126 | [0.069,0.228] |
| Position*** | 1.388 | 0.133 | 10.472 | <0.001 | 4.006 | [3.09,5.195] |
| R2byR3*** | -1.667 | 0.131 | -12.679 | <0.001 | 0.189 | [0.146,0.244] |
| Language* | 1.283 | 0.607 | 2.112 | 0.035 | 3.606 | [1.097,11.857] |
| Who places | 0.084 | 0.126 | 0.667 | 0.505 | 1.088 | [0.849,1.394] |
| Position×R2byR3*** | 1.395 | 0.258 | 5.403 | <0.001 | 4.034 | [2.432,6.691] |
| Position×Language*** | 2.481 | 0.265 | 9.361 | <0.001 | 11.957 | [7.112,20.103] |
| Position×Who places | 0.106 | 0.252 | 0.421 | 0.674 | 1.112 | [0.678,1.824] |
| R2byR3×Language* | -0.558 | 0.263 | -2.124 | 0.034 | 0.572 | [0.342,0.958] |
| R2byR3×Who places | 0.037 | 0.252 | 0.145 | 0.885 | 1.037 | [0.633,1.701] |
| Language×Who places** | -0.775 | 0.253 | -3.069 | 0.002 | 0.461 | [0.281,0.756] |
| Position×R2byR3×Language | 0.423 | 0.516 | 0.819 | 0.413 | 1.526 | [0.555,4.199] |
| Position×R2byR3×Who places | 0.042 | 0.505 | 0.083 | 0.934 | 1.043 | [0.388,2.805] |
| Position×Language×Who places | 0.488 | 0.505 | 0.966 | 0.334 | 1.628 | [0.605,4.379] |
| R2byR3×Language×Who places | -0.380 | 0.505 | -0.753 | 0.451 | 0.684 | [0.254,1.839] |
| Position×R2byR3×Language×Who places | 1.537 | 1.009 | 1.522 | 0.128 | 4.648 | [0.643,33.617] |
| Significance Codes: <.001 '***'; <.01 '**'; <.05 '*' | | | | | | |

While there was an interaction between language and who places (an effect is present in English, but not in Japanese), there were no other interactions with the who places-variable. This suggests this is a separate effect of interacting with an object, which replicates previous effects (cf., Coventry et al., 2008, 2014, experiment 1), showing that when participants place the object themselves there is a higher likelihood of *this-*use in English, but an opposite effect in Japanese. However, there is no other effect with who places, so while this effect could be followed up on in a future publication, it does not affect the main findings presented in the paper. For transparency we include it in these Supplementary Materials, to not distract from the main narrative.

# References

Coventry, K. R., Griffiths, D., & Hamilton, C. J. (2014). Spatial demonstratives and perceptual space: Describing and remembering object location. *Cognitive Psychology*, *69*, 46–70. https://doi.org/10.1016/j.cogpsych.2013.12.001

Coventry, K. R., Valdés, B., Castillo, A., & Guijarro-Fuentes, P. (2008). Language within your reach: Near-far perceptual space and spatial demonstratives. *Cognition*, *108*(3), 889–895. https://doi.org/10.1016/j.cognition.2008.06.010
